# The diversity of microparasites of rodents: a comparative analysis that helps in identifying rodent-borne rich habitats in Southeast Asia

**DOI:** 10.3402/iee.v3i0.20178

**Published:** 2013-04-08

**Authors:** Frédéric Bordes, Vincent Herbreteau, Stéphane Dupuy, Yannick Chaval, Annelise Tran, Serge Morand

**Affiliations:** 1 Institut des Sciences de l'Evolution, CNRS-IRD-UM2, Université de Montpellier 2, Montpellier, France; 2 ESPACE-DEV-IRD, Université de Montpellier 2, Montpellier, France; 3 CIRAD, UMR TETIS, Montpellier, France; 4 Centre de Biologie et de Gestion des Population, INRA, Montferrier sur Lez, France; 5 CIRAD, UR AGIRs, Montpellier, France; 6 Department of Parasitology, Faculty of Veterinary Sciences, Kasetsart University, Bangkok, Thailand

**Keywords:** rodent-borne diseases, landscape, transmission ecology, comparative analysis

## Abstract

**Background:**

Predicting habitats prone to favor disease transmission is challenging due to confounding information on habitats, reservoirs, and diseases. Comparative analysis, which aims at investigating ecological and evolutionary patterns among species, is a tool that may help. The emergence of zoonotic pathogens is a major health concern and is closely linked to habitat modifications by human activities. Risk assessment requires a better knowledge of the interactions between hosts, parasites, and the landscape.

**Methods:**

We used information from a field spatial study that investigated the distribution of murid rodents, in various habitats of three countries in Southeast Asia, in combination with their status of infection by 10 taxa of microparasites obtained from the literature. Microparasite species richness was calculated by rodent species on 20,272 rodents of 13 species. Regression tree models and generalized linear models were used to explain microparasite diversity by the average distance between the trapping site and five categories of land cover: forest, steep agriculture land, flat agriculture land, water, and built-up surfaces. Another variable taken into account was the slope.

**Results:**

We found that microparasite diversity was positively associated with flat agriculture land, in this context mainly rice fields, and negatively associated with slope. Microparasite diversity decreased sharply a 100 m or less from flat agriculture land.

**Conclusion:**

We conclude that there is high microparasite circulation in rodents of flooded farmlands, meaning possibly a higher risk of disease for human inhabitants.

All attempts to understand disease ecology and parasite transmission have to consider often, if not always, two important factors. First, hosts (at species or individual levels) are not equal when it comes to parasite transmission, i.e. a great heterogeneity exists in parasite transmission with some individuals (or species) being responsible for a disproportionate number of transmission events (1, 2). This heterogeneity can be related to differences in susceptibility (3) and/or in exposure to infected hosts or environment (2). Second, across space, another heterogeneity is observed with some habitats or landscapes prone to differentially affect parasitic or vectors persistence or transmission between hosts (4–6). This heterogeneity may reflect biodiversity change and sometimes biodiversity loss, which may affect reservoir species composition (7–10).

The influence of landscape heterogeneity on disease ecology gains more and more importance when there are accelerated environmental changes, such as deforestation for agricultural purposes. All these environmental changes are prone to affect the location and densities of parasites, hosts, or vectors (11–13). Consequently, these environmental changes can affect positively or negatively parasite transmission as emphasized by Ostfeld et al. (7). Assuming that hosts and environments may contribute disproportionately to parasite transmission, the challenge is to identify their links to predict disease persistence or emergence (2).

From this perspective, rodent-borne diseases caused by major pathogens like *Leptospira* sp., hantaviruses, arenaviruses, *Borrelia* sp. (agents of Lyme disease), *Yersinia pestis* (agent of Plague), or *Bartonella* sp. have, for a long time, been probed so as to identify their rodent reservoirs, and more precisely as emphasized by Haydon et al. (14) their reservoir complexes, i.e. heterogeneity in host species, composition, and importance (14, 15) and/or transmission places, i.e. heterogeneity in space (16, 17).

Recently, two concepts have emerged: ‘synanthropic species’ (namely species ecologically associated with humans) and ‘generalist’ species (i.e. prone to live in peridomestic habitats or to invade disturbed habitats) (16, 18, 19) – information on preferred habitats of Southeast Asian rodents is available on www.ceropath.org. For example, outbreaks of Hantavirus pulmonary syndrome in Panama have been positively correlated to species-poor rodent communities in disturbed habitats and to the dominance of two reservoir hosts for Hantavirus (*Oligoryzomys fulvescens* and *Zygodontomys brevicauda)* in such areas (18, 20). Interestingly, such a trend was recently confirmed by a comparative study performed by McFarlane et al. (19) in the Asian-Australian region, in which they found that wild mammal hosts (mainly rodents and bats) of zoonotic emerging infectious diseases are 15 times more likely to inhabit human-modified environments. In other words, deforestation and other disturbances are supposed to increase the distribution and abundance of generalist rodent species, which are prone to being reservoirs of human pathogens.

However, we have also to consider that most places in tropical areas are already largely human-modified, even supposed preserved habitats such as forests, especially in Southeast Asia (SEA) (21). The real challenge is rather to identify specific environmental determinants likely to explain higher rodent parasite infections in disturbed areas: explaining the spatial heterogeneity of infection patterns is the main objective of spatial epidemiology (4). Moreover and importantly, this goal could be more significant than tracking host species reservoirs *per se* for at least three reasons. First, identification of ‘reservoirs’ is not always easy as both the detection of antibodies and direct detection of pathogens in wild hosts are difficult to perform and to interpret (14). Second, if some associations between a particular disease in humans and the presence of a given host reservoir species exist, infection in humans is not always congruent with distribution of host reservoir species. For example, the bank vole is the most widespread and abundant rodent species in Europe and the main reservoir of Puumala virus, the agent of the hemorrhagic fever with renal syndrome (HFRS) in humans, but the cases of HFRS are restricted to a limited portion of its global distribution (17). The spatial distribution of this Hantavirus further depends on parameters such as forest patch size and connectivity of the most suitable rodent habitats (22), or on the optimal conditions for the survival of the virus outside the host. Third, identifying a reservoir for only one parasite may be, at least in some areas, rather restrictive due to the important circulation of multiple pathogens in natural systems, especially in rodents (23, 24). In fact, multiple infection, or concomitant infection, is the rule and only starts to be considered as a key factor in natural wild systems, due to parasite species interactions or impacts related to multiple infections on hosts (24–26). As a result, parasitic risk is global, with many parasite species liable to infect humans.

Comparative analyses seek at identifying host determinants, ecological or life traits, of parasite diversity. Few studies have investigated environmental niches or habitat characteristics as potential determinants of the parasite species richness, often because of lack of accurate information on hosts’ habitats (27). Moreover, a difficulty is linked to the fact that the parasite diversity observed in a given individual host is always lower than the parasite diversity at the host population level, which is also lower than the parasite diversity at the host species level. This means that trying to relate the parasite diversity observed at the host individual level with the surrounding features of landscape may hardly help at identifying pathogenic landscape for multiple diseases.

Focusing on rodent-borne diseases in SEA, we aimed to identify habitat of high richness in rodent-borne diseases using a comparative analysis approach. For this, we crossed a dataset on microparasite diversity (agents of rodent-borne diseases) in murid rodents in SEA, a major clade of reservoirs of zoonotic diseases (15), with an original geo-referenced dataset in order to detect association between landscape features, where rodents were trapped, and the total extant of microparasite species richness harbored by rodent species. More precisely, we used the land covers of seven sites in Thailand, Cambodia, and Lao PDR (28) and literature data on the diversity of microbial agents circulating in rodents of these countries (29) to identify pathogenic habitats in this tropical area.

## Material and methods

### Rodents

Rodents were trapped in the Cambodian provinces of Preah Sihanouk and Mondulkiri, the Thai provinces of Loei, Buriram and Nan, and the Lao provinces of Champasak and Luang Prabang (see www.ceropath.org).

These locations represent a variety of habitats, in relation to human pressures and land usage. Habitats were ranked as: 1) forests and mature plantations, 2) non-flooded lands or fields (shrubby wasteland, young plantations, orchards), 3) rain-fed lowland paddy rice fields (cultivated floodplain), 4) households (in villages or city). Each natural and agricultural habitat was sampled with an equal pressure using a stratified trapping protocol. For each trapping session, 30 trap lines of 10 locally made cage-traps (separated at 5 m intervals) were deployed during four nights. The trapping pressure could be estimated at 1,200 trap night for each locality at each season. Villages and isolated houses, which correspond to the fourth habitat category, were also sampled using cage-traps distributed to residents. Additional trappings were obtained using local hunters, from where less accurate precision in the trapping sites were often recorded.

Geographical coordinates of trap line devices and households were systematically recorded with a GPS and the surrounding landscape was described by field observation with a three-level classification: ‘low resolution’ for the main landscape categories (forest, non-flooded agriculture fields, irrigated/rain-fed agriculture field, settlement), ‘medium resolution’ for a more detailed category nested in the ‘low resolution’ (for example: isolated farm in ‘settlement’, rice field in ‘rain-fed agriculture field’, corn field in ‘non-flooded agriculture field’, dry evergreen in ‘forest’) and ‘high resolution’ nested ‘medium resolution’ to give more precision (harvested rice field, inside rice store, etc.).

The accuracy of geographical coordinates ranges from: 1 (less than 10 m, i.e. the precision of GPS) for geographical coordinates taken at the individual trap; 2 (less than 100 m, i.e. a trap line of ten traps is less than 100 m long) for geographical coordinates taken in the middle of traps’ line; and 3 (less than 1,000 m, i.e. a rodent trapped in a given field or in a given village) for geographical coordinates for a rodent trapped by hunter in an area around these coordinates.

Two trapping sessions were realized per locality during different seasons from 2008 to 2009. Pictures, habitat description and coordinates of trap lines are available in the ‘research/study’ areas and ‘research/protocols’ sections of the CERoPath project web site (www.ceropath.org).

This standardized and structured trapping protocol helps at minimizing biases when comparing within and between sites, which has permitted to compare the prevalence of infection of rodents by bacteria of the genus *Leptospira* in the two sites of Cambodia (30). It was also designed to have an estimation of the rodent density using the number of catches by night trap.

Rodents were identified on the basis of their morphology or using species primer specific and/or barcoding assignment. Complete data for animals used as reference for barcoding assignment are available on the ‘Barcoding Tool/RodentSEA’ section of the CERoPath project web site: http://www.ceropath.org/.

Overall, 2,427 murine rodents trapped in the seven study sites were integrated into a Geographic Information System (GIS) based on the geographic coordinates of their sampling site. They represent a total of 30 species, but include 12 species with less than 10 specimens.

### Environmental indices

For each site, recent 2007–2008 high spatial resolution SPOT satellite images were acquired. When possible, cloud-free scenes (i.e. from the dry season) were chosen. The scenes had a pixel size of 2.5 m in panchromatic mode and 10 m in multispectral mode. SPOT-Digital Elevation Model (DEM) with a spatial resolution of 20 m together with the SRTM (Shuttle Radar Topography Mission, http://srtm.usgs.gov/) DEM (90 m resolution) was also acquired. These DEMs allow several calculations to describe the topography, including the slope and the delineation of watersheds.

For each site the SPOT scene was classified into different land-cover types using an object-based approach (eCognition Developer^®^ commercial software). Each scene was first segmented into objects which were then classified using a supervised process based on three types of object properties: intrinsic characteristics (reflectance values, slope values, shape and texture), topologic characteristics (relations to neighboring objects) and contextual characteristics (semantic relationships between objects). They were merged into five main classes: forest, steep agriculture land, flat agriculture land, water, and built-up surfaces that are present in the seven study sites. Classification accuracy was assessed by field observations and photo interpretation using Google Earth^®^ – see (24) for more information. The land-cover maps of the seven sites can be visualized at www.ceropath.org.

The land-cover maps and the DEM were integrated into a GIS in order to compute some landscape metrics for each trapping site. As we are interested for this comparative analysis at indentifying preferred habitats of rodent species, and not the structure of the landscape, we computed minimal distances between each individual and each land-cover type (only for those having a precise geographic location of accuracy 1 and 2): distance to forests, distance to steep agriculture lands (i.e. non-flooded), distance to flat agriculture lands (i.e. flooded, irrigated, paddy fields), distance to built zones (i.e. villages, cities), distance to water areas (i.e. ponds, lakes, rivers), elevation and slope.

The mean values of these metrics were then calculated for each species of rodents (with accuracy values of 1 or 2) (Table 1).

**Table 1:** Average attributes of spatial indices (in meter) for the rodent species trapped in seven localities of Southeast Asia (Thailand, Lao PDR and Cambodia)

Species	Elevation	Slope	Distance to forest	Distance to steep agriculture	Distance to flat agriculture	Distance to built area	Distance to water	Total rodents (accuracy 1 and 2)	Total trapped rodents (accuracy 1, 2 and 3)
*Bandicota indica*	227	2	57	366	32	241	955	127	127
*Bandicota savilei*	178	2	150	1126	8	588	2502	72	131
*Berylmys berdmorei*	126	5	23	348	85	232	833	26	36
*Maxomys surifer*	79	6	15	243	54	247	1493	78	130
*Mus caroli*	310	4	76	193	104	375	2178	110	115
*Mus cervicolor*	263	3	197	547	271	198	512	82	102
*Mus cookii*	343	6	68	94	119	234	3124	141	197
*Niviventer fulvescens*	187	5	18	514	70	274	1797	75	75
*Rattus argentiventer*	11	2	366	1414	39	58	281	12	160
*Rattus exulans*	139	2	110	1216	42	192	1184	226	509
*Rattus losea*	291	4	66	193	76	586	1911	90	110
*Rattus norvegicus*	8	2	175	942	72	9	185	17	25
*Rattus tanezumi*	231	3	72	511	56	115	1314	219	353

### Parasite diversity

We compiled surveys of microparasites investigated in rodents trapped in SEA and published in the literature (29). The data comprise a total of 20,272 rodents from 13 species of murine rodents that have been investigated for a total of 10 important microparasites’ taxa (Table 2). This microparasite dataset helped at obtaining the overall microparasite diversity of rodents. Therefore, it does not concern the same rodent individuals as those trapped by the CERoPath project, which has only investigated a few microparasites, which are incorporated in this dataset (30, 31) (but see www.ceropath.org). The microparasites were viruses, bacteria and protozoans. Viruses were all zoonotic and included hantaviruses, Lymphocytic choriomeningitis virus (family Arenaviridae, genus Arenavirus), Rabies virus, and Hepatitis E virus. The bacteria were also all zoonotic and concerned *Leptopsira spp*. agents of leptospirosis, *Bartonella spp*. agents of bartonellosis and *Orientia tsutsugamushi*, the agent of Scrub Typhus. *Bartonella sp*. and *Orientia tsutsugamushi* are arthropod- borne agents, whereas *Leptospira spp*. are indirectly transmitted via contact with water or soils contaminated by urine of infected rodents. Finally, protozoans investigated were *Toxoplasma gondii* and *Babesia spp*., both also zoonotic, notably for *Toxoplasma gondii*. Microparasite richness was defined as the number of pathogen species for which each rodent species was found positive.

**Table 2:** Survey of infection by microparasites (viruses, bacteria, protozoans) of rodent species in Thailand, Lao PDR and Cambodia, with number of investigated individuals for each species [see references in supplementary materials of Herbreteau et al. (29) with additional data from Angelakis et al. 2009 (48), Bai et al. 2009 (49), Ivanova et al. (30), Jiyipong et al. (31)]

Species	*Leptospira*	*Orientia spp*	*Bartonella spp*	Hantavirus	Herpes virus	LCM virus	Rabies virus	*Toxoplasma gondii*	*Trypanosoma spp*	*Babesia spp*.	Total microparasites	Total rodents
*Bandicota indica*	1	1	1	1	1	1	0	1	1	1	9	3839
*Bandicota savilei*	1	1	1	1	1	1	0	0	1	–	7	1176
*Berylmys berdmorei*	1	1	1	0			0	0	1	–	4	117
*Maxomys surifer*	1	0	1	0	–	–	1	1	1	–	5	464
*Mus caroli*	0	0	1	0	0	0	–	–	–	–	1	211
*Mus cervicolor*	0	0	1	0	–	–	–	0	0	–	1	256
*Mus cookii*	–	–	1	0	–	–	–	0	0	–	1	148
*Niviventer fulvescens*	1	1	1	0	–	–	0	0	0	–	3	126
*Rattus argentiventer*	1	1	1	0	1	1			1	–	6	534
*Rattus exulans*	1	1	1	1	0	1	0	1	1	0	7	3289
*Rattus losea*	1	1	1	1	0	0	0	0	1	–	5	899
*Rattus norvegicus*	1	1	1	1	1	1	0	0	1	–	7	1405
*Rattus tanezumi*	1	1	1	1	1	1	0	1	1	–	8	7808
Total rodents												20272

### Statistical analysis

We performed a principal components analysis (PCA) on individual number of rodent species trapped in each the four types of habitat (i.e. low resolution) to illustrate their distributions using the package ‘ade4’ in R software (R Development Core Team, 2010).

In order to better estimate the preferred habitat of rodent species, we performed tree regression analysis (TRA) (32, 33) on the minimal distances between each individual rodent and each land-cover habitat type using package tree in the R software (R Development Core Team, 2010).

We performed generalized linear models (GLM) to identify the likely variables that may explain the microbial diversity of rodents using the R software (R Development Core Team, 2010). We performed a multiple regression with microparasite species richness as the dependent variable and environmental indices as the independent variables: distance to forest, distance to steep agriculture land, distance to flat agriculture land, distance to built-up zones, distance to water, and slope (which refers to each individual rodent trap). We selected the models using a backward procedure and the Akaike Information Criteria (AIC) to identify the minimal adequate model. As colinearity was found high between some of explanatory variables, we selected among these associated variables by testing them successively, retaining the variables providing the lowest AIC.

We conducted analysis on raw data for the 13 rodent species for which we have obtained average estimates of their distribution according to the above spatial indices (Table 1) and that have been investigated sufficiently for microparasite richness (Table 2).

We compared the results of the GLM with a tree regression analysis on the same independent variables to explained microparasite species richness.

### Phylogenetic test

Two or more rodent species may share similar microparasite species richness, and potentially the same microparasite species, because they have inherited them from a common ancestor and/or because they have co-evolved with certain species of macroparasites. This long-term co-evolution may explain patterns of specificity (such as hantaviruses with some Rattini hosts). Co-evolutionary relationships may be then more important than actual ecology at shaping the diversity and richness of microparasites. To avoid these phylogenetic influences when investigating patterns of parasite species richness, we tested our predictions using the independent contrasts method (34). The phylogeny of rodents follows the recent study of Pages et al. (35) on SEA murids. Contrasts were calculated using Ape (36) implemented in R (R Development Core Team, 2010). Contrasts were then analyzed with all correlations between contrasts forced through the origin (37). We conducted the phylogenetic test on the model selected using the raw data.

## Results

**Fig. 1:**
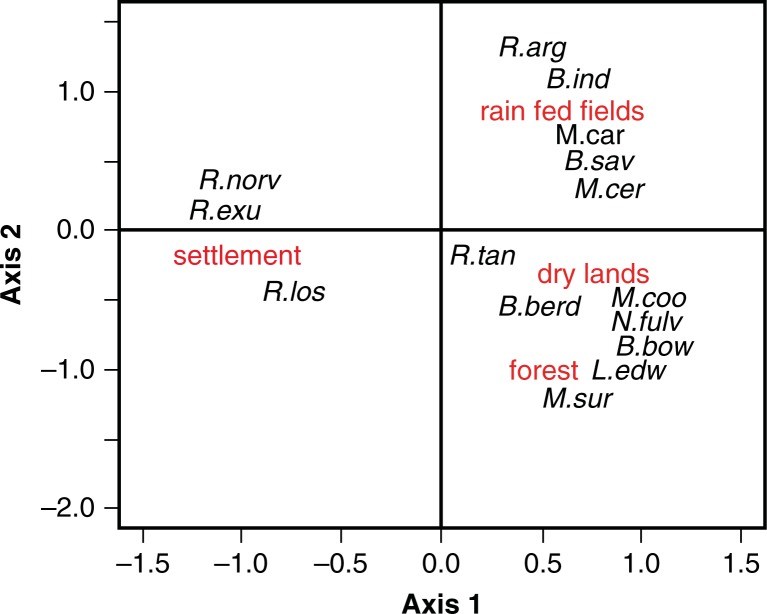
Distribution of rodent species according to habitat types: paddy fields (lowland rain-fed), non-flooded lands, forests, households and settlement) on the two first axes of a principal component analysis. The axis 1 and 2 accounted for 85% of the variance. (*B.ind: Bandicota; B.sav: Bandicota savilei; B.berd: Berrylmys berdmorei; B.bow: Berrylmys bowersi; L.edw: Leopodamys edwarsi; M.sur: Maxomys surifer; M.car: Mus caroli; M.cer: Mus cervicolor; M.coo: Mus cooki; N.fulv: Niviventer fulvescens; R.arg: Rattus argentiventer; R.exu: Rattus exulans; R.los*=*Rattus losea; R.norv=Rattus norvegcius; R.tan*=*R. tanezumi*).

PCA on an individual number of rodent species trapped in each the four types of habitat showed that the first two axes accounted for most of the total variability in the data set. The first axis explained 46.5% of the variability, and the second axis explained 39% of the variability (Fig. 1). Habitat preference, as this level of characterization, seems to be the case for some of these rodent species. *Rattus exulans* and *R. norvegicus* were found mainly in household. Some other species showed a strong preference for rain-fed paddy fields (*Bandicota indica, R. argentiventer*) or forests (*Leopoldamys edwardsi, Maxomys surifer*). However some rodent species show preference for two habitats, such as *Mus cervicolor* in flooded and non-flooded fields. *Rattus tanezumi* is the one that shows no preference and that can be found in all of the four habitats.

**Fig. 2:**
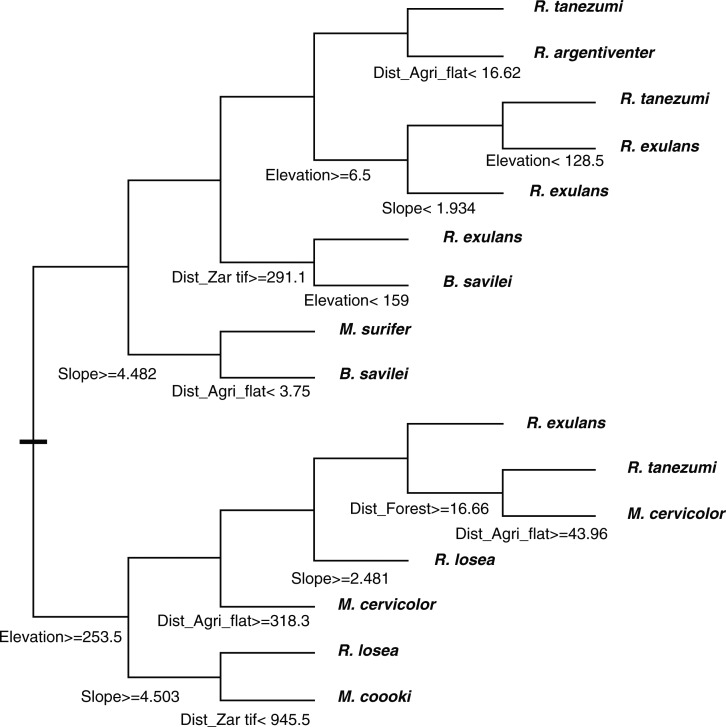
Regression tree model explaining distribution of rodents in relation to distance to main habitats: forest, steep agriculture, flat agriculture, settlement, and with slope and elevation.

Tree regression analysis allowed a better characterization of the distribution of several rodent species in relation to the minimum distance to each of the main habitats, slope, and elevation (Fig. 2). In particular, *R. tanezumi* and the three species of *Mus* were confirmed to occur in various habitats. But, the main striking result is the ubiquitous distribution of *Rattus exulans*, which may be found in isolated households or small settlements for almost all kinds of habitat.

**Fig. 3:**
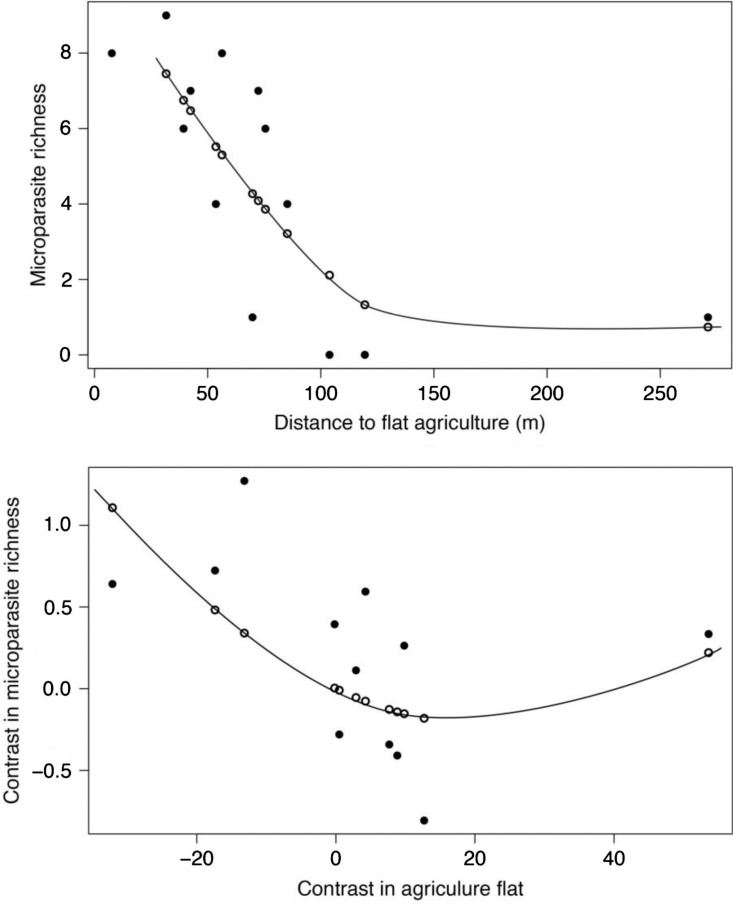
Relationship between microparasite species richness and distance to flat agriculture (i.e. irrigated/flooded, paddy rice fields) (A) using raw data (the distribution is fitted to a polynomial regression of second order, R2=0.63, F2,11=8.50, P=0.007) and (B) using independent contrasts (the distribution is fitted to a polynomial regression of second order without intercept, R2=0.41, F2,10=3.40, P=0.07).

We conducted analysis on raw data for the 13 rodent species and found that the best model, using the AIC criterion, showed that microparasite species richness was negatively correlated with distance to flat agriculture area (i.e. paddy fields) and negatively to the slope (Table 3). Microparasite species richness was found related to rodents trapped near flat agriculture area. Moreover, the microparasite species richness decreased sharply at less than 100 m (Fig. 3)A.

**Table 3:** Best model explaining microparasite richness in rodents in relation to habitat indices (initial model with distance to forest, distance to steep agriculture, distance to flat agriculture, distance to water, slope, sample size) (AIC=56.94) (with SD=standard deviation of the slope, P=probability)

Independent variables	Slope (SD, P)	*F*-test (P)	R^2^, *F*-total (P)
Distance to flat agriculture	−0.03 (0.008)	27.56 (0.007)	R^2^=0.74 F_2,10_=14.3
Slope	−1.23 (0.35)	49.92 (0.005)	(0.001)

We obtained similar results using tree regression analysis with a distance from flat agriculture <63 m characterized by a high microparasite species richness (7.0 in mean), and a distance from flat agriculture >63 m characterized by a microparasite species richness (2.7 in mean).

The analysis on the independent contrasts of agriculture flat and microparasite species richness established that microparasite species richness was found also related to flat agriculture area. However, a slight increase of microparasite species richness was observed in rodents found far from flat agricultural areas (Fig. 3B).

## Discussion

### Distribution of rodents in habitat

This study has permitted to improve the knowledge on habitat preference of SEA rodents. Although the principal component analysis on rodent occurrence in the main habitats gave similar results as previously obtained in the sites of Cambodia (30), the TR and GLM models improved our knowledge on the environmental niches of these rodents using land covers and geo-referencing to main habitats. Interestingly, if we confirm the habitat specificity for some species, such as *M. surifer* in forest or *R. norvigicus* in houses, the results of these models suggest large habitat range for species like *R. tanezumi, M. cooki* and even more surprisingly for *R. exulans*. This later species is mostly restricted to houses and the surrounding area but can also be found in almost every isolated household or small settlement within any kind of surrounding habitat.

### High microparasite diversity in flat agriculture areas

Most of studies related to rodent-borne diseases to date focused on one pathogen, notably hantaviruses or arenaviruses, trying to link habitat characteristics, structure of rodent communities or season to prevalence of infections in rodents or to human outbreaks. The main limit of such studies is that they ignore the great diversity of pathogens circulating in rodents, particularly in the tropical areas where microparasite diversity is higher compared to the temperate zones (38). Our comparative analysis using two important datasets (i.e. 1,275 of 2,070 rodents for the computation of environmental indices and more than 20,000 rodents for the estimation of microbial diversity) is one of the few that questions the environmental determinants of parasite diversity in tropical area. Both TR and GLM models showed similar results with higher microparasite species richness that can be harbored in rodents trapped close to flat agriculture fields. This comparative analysis allows us to infer that higher microparasite diversity in rodents is found in agricultural lands in flat or low-slope areas in SEA. Moreover, and importantly, it seems that a threshold is observed (Fig. 3A). Beyond 100 m a sharp decrease in microparasite diversity was observed. Clearly, this suggests that rodents with an environmental niche away from irrigated/flooded rice fields, according to their geo-localized distribution in the seven studied localities, harboring potentially less microparasites species, may poorly participate in parasite transmission. This result is very intriguing as it is usually expected that forests are the more favorable habitats to insure parasite transmission as observed in different vector-borne disease systems (39, 40). However, other studies that focused on helminth parasitism have led to contrasted results with higher parasite species richness in disturbed habitats or logged forests for some worms but not for others (41).

On the contrary, our results rather sustain the emerging pattern that hosts living in human-modified habitats may harbor higher parasite loads and/or that vectors may be more abundant in such areas (11, 13, 18, 42, 43). Moreover, the negative correlation between parasite diversity and slope stresses that some factors in cultivated and often flooded lowlands, notably rice fields, are extremely favorable to parasite transmission.

Interestingly, using the independent contrasts method, a slight increase in microparasite species richness in rodents was observed when moving far from agriculture in flat area. This could be related to more ‘natural conditions’ prone to also favor higher parasite diversity as observed, for example, for helminths in rodents (41).

### Should health monitoring continue to focus on rice fields?

Various works established the great biodiversity associated with the rice field agro-system in Asian countries – see (44) for a review. Because important resources are available for various species, rice fields may then be important foraging areas for rodents. This fact is largely sustained by the role of rodents as pests in agriculture in Asia, notably in rice fields where every year they consume food that could feed 200 million people for an entire year (45). Foraging areas are ideal places for parasite transmission due to important host densities promoting direct or indirect intra- or interspecific interactions. Moreover, due to high arthropod diversity in rice fields (44) and high rodent densities, we may expect enhanced encounters or exchanges with multiple vectors such as ticks or mites (46). Finally, persistence of water in flat and flooded areas may be an important factor to insure parasite persistence in the environment, notably for *Leptospira* (30).

However, rice fields are historical elements of SEA landscapes, which are more concerned today by major changes such as increased deforestation or urbanization. The other main problem that faces SEA countries is associated to the changes in climate variability such as rainfall patterns (monsoon) by increasing risks of disease outbreaks linked to heavy rainfalls and extreme floods (47).

## Conclusion

This study improves our knowledge on the distribution of rodents in SEA and particularly the synanthropic rodents such as *R. tanezumi* and *R. exulans*, which showed low habitat specificity using geo-referenced trapped position in land covers. *Rattus exulans* even if mostly restricted to households can be found in every isolated small settlement within any kind of surrounding habitat. The comparative analyses using either GLM or TR models showed that microparasites species-rich rodents were found near flat agriculture fields (i.e. paddy fields) suggesting that this habitat may favor microparasite transmission and should be targeted for rodent-borne disease surveillance. Future studies should investigate local microparasite diversity at small scales by taking into account the structure of the landscape (i.e. habitat diversity and fragmentation).
